# Korean Red Ginseng Saponin Fraction Rich in Ginsenoside-Rb1, Rc and Rb2 Attenuates the Severity of Mouse Collagen-Induced Arthritis

**DOI:** 10.1155/2014/748964

**Published:** 2014-04-16

**Authors:** Mehari Endale, Eun Ju Im, Joo Young Lee, Sung Dae Kim, Taddesse Yayeh, Yong-Bum Song, Yi-Seong Kwak, Chaekyun Kim, Seung-Hyung Kim, Seong-Soo Roh, Jae Youl Cho, Man Hee Rhee

**Affiliations:** ^1^Laboratory of Veterinary Physiology and Cell Signaling, College of Veterinary Medicine, Kyungpook National University, Daegu 702-701, Republic of Korea; ^2^Division of Immunobiology, Cincinnati Children's Hospital Medical Center, Cincinnati, OH 45219, USA; ^3^Research Center, Dongnam Institute of Radiological & Medical Sciences, Busan 619-953, Republic of Korea; ^4^Debre Markos University, Debre Markos, Ethiopia; ^5^Ginseng Corporation Central Research Institute, Daejeon 305-805, Republic of Korea; ^6^Department of Pharmacology, Inha University School of Medicine, Incheon 400-712, Republic of Korea; ^7^Institute of Traditional Medicine & Bioscience, Daejeon University, Daejeon 300-716, Republic of Korea; ^8^Department of Herbology, College of Korean Medicine, Daegu Hanny University, Gyeongsan 712-715, Republic of Korea; ^9^Department of Genetic Engineering, Sungkyunkwan University, Suwon 440-746, Republic of Korea

## Abstract

Despite a multitude of reports on anti-inflammatory properties of ginseng extracts or individual ginsenosides, data on antiarthritic effect of ginseng saponin preparation with mixed ginsenosides is limited. On the other hand, a combined therapy of safe and inexpensive plant-derived natural products such as ginsenosides can be considered as an alternative to treat arthritis. Our previous * in vitro* data displayed a strong anti-inflammatory action of red ginseng saponin fraction-A (RGSF-A). We, herein, report a marked antiarthritic property of RGSF-A rich in ginsenoside Rb1, Rc, and Rb2. Collagen-induced arthritic (CIA) mice were treated with RGSF-A or methotrexate (MTX) for 5 weeks. Joint pathology, serum antibody production and leukocye activation, cytokine production in the circulation, lymph nodes, and joints were examined. RGSF-A markedly reduced severity of arthritis, cellular infiltration, and cartilage damage. It suppressed CD3^+^/CD69^+^, CD4^+^/CD25^+^, CD8^+^ T-cell, CD19^+^, B220/CD23^+^ B-cell, MHCII^+^/CD11c^+^, and Gr-1^+^/CD11b^+^ cell activations. It further suppressed anti-CII- or anti-RF-IgG/IgM, TNF-**α**, IL-1**β**, IL-17, and IL-6 secretions but stimulated IL-10 levels in the serum, joint, or splenocyte. RGSF-A attenuated arthritis severity, modified leukocyte activations, and restored cytokine imbalances, suggesting that it can be considered as an antiarthritic agent with the capacity to ameliorate the immune and inflammatory responses in CIA mice.

## 1. Introduction

Rheumatoid arthritis (RA) is a chronic, inflammatory, and systemic autoimmune disease of diarthrodial joints which affects about 1% of the general population [1]. The initiation and establishment of RA is the first stage of the disease, which begins with unregulated immunoinflammatory cell recruitment, activation, and retention in draining lymph nodes (dLN) and affected joints accompanied by the second stage of inflammatory responses such as synovial hyperplasia, pannus formation, cartilage, and bone erosion [2]. Together with the indicated stages of responses, autoantibody, cytokine, and matrix metalloproteinase (MMP) productions perpetuate the disease progression [3].

The main cells involved in RA pathogenesis are T- and B-lymphocytes, dendritic cell, endothelial cells, osteoclast, synoviocytes, and chondrocyte for which no one cell type but interactions of all define the disease pathology of RA [4]. Unregulated interactions of these cells create imbalances between proinflammatory and anti-inflammatory cytokine activities that favor the induction of autoimmunity, chronic inflammation, and joint damage [3]. Thus, agents that inhibit the former and stimulate the later are considered as candidate therapies of RA [5]. Given the various immune cell interactions involved in cell trafficking and recruitment to inflamed joints, identifying new agents that normalize such interactions as well as reducing cellular influx and joint inflammation is an important strategy for RA therapy.

Type II collagen-induced arthritis (CIA) is an animal model of rheumatoid arthritis that has been used extensively to address questions of disease pathogenesis. Susceptibility to CIA is strongly associated with major histocompatibility complex class II genes, and the development of arthritis is accompanied by a robust T- and B-cells response to type-II collagen [6]. Both proinflammatory and anti-inflammatory cytokines are common features of the arthritic joints of murine CIA. And, the main pathological features of CIA include proliferative synovitis with infiltration of inflammatory cells, synovial hyperplasia (pannus formation), cartilage degradation, bone erosion, and fibrosis. Since CIA shares clinical, histological, and immunological features with RA and has been used to validate novel therapeutic targets [7], we examine the potential of our test compound against collagen-induced arthritis in mice.

Different treatment strategies ranging from conventional to biological therapies have been utilized for RA treatment. However, each strategy is reported to be associated with certain side effects. For example, among the conventional therapies, the immunosuppressive disease-modifying antirheumatic drugs (DMARDs) cause toxicity under chronic treatments [8], or patients become refractory to therapy [9]. In addition, although biological therapies showed great advances in RA treatment, they are reported to be associated with high cost, limited remission, hypersensitivity to medications, and risk of infection [8, 10]. Thus, further advances in identifying new entities that could treat arthritis while minimizing side effects are required.

On the other hand, herbal medicines that possess anti-inflammatory properties with minimal side effects have nowadays received much attention and are recommended as natural alternative agents against arthritis. Recent studies reported that natural products including flavonoids, catechins, and terpenes exhibit antiarthritic effects [11]. For example, pomegranate extract [12], epigallocatechin-3-gallate [13], resveratrol [14], curcumin [15], and others [11] have been reported to suppress inflammatory and catabolic molecular mediators of arthritis.

Ginseng from the root of* Panax ginseng* C. A. Meyer is a widespread herbal medicine that still occupies a prominent position in the list of best-selling natural products and is considered as the most widely taken herbal product in the world [16, 17]. Ginseng's potential therapeutic effects have been attributed to its immunomodulatory, antioxidant, and anti-inflammatory activities [16, 18, 19]. The anti-inflammatory effects of ginseng extracts and ginsenosides are associated with their properties of cytokine regulation, immune cell phagocytosis, and modulation of T- and B-lymphocytes activations [20, 21]. Recent studies have further shown the anti-inflammatory effects of ginseng extracts and ginsenosides in cellular responses triggered by different inducers including endotoxin, tumor necrosis factor-*α* (TNF-*α*), interferon-*γ* (INF-*γ*), and other stimuli [16, 21, 22].

Saponin components of ginsenosides are divided into two different structural classes: the 20(*S*)-protopanaxadiol (PD) group of ginsenosides including Ra1, Ra2, Ra3, Rb1, Rb2, Rb3, Rc, Rd, Rg3, and Rh2 and 20(*S*)-protopanaxatriol (PT) group ginsenosides such as Re, Rf, Rg1, Rg2, and Rh1 [23]. PD ginsenosides including Rb1 [24], Rd [25], Rg3 [26], and Rh2 [27] and PT ginsenosides such as Rg1 [28, 29] and Rh1 [30] have been reported to exhibit both* in vivo* and* in vitro* anti-inflammatory properties in different studies related to inflammation. Individual ginsenoside, however, due to incomplete pharmacokinetic parameters and unknown toxicities, has rarely been reported for the clinical study so far [31].

**Figure 1:**
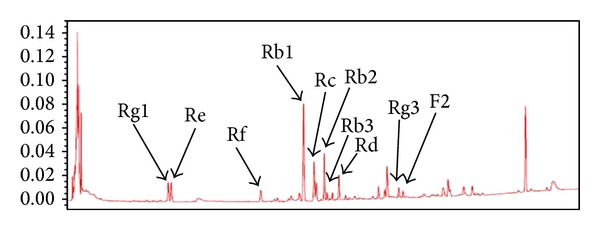
HPLC chromatographic profile of red ginseng saponin fraction-A (RGSF-A).

On the other hand, ginseng saponin components are commonly taken as a remedy in a combined form in herbal preparations. Modifying the proportions of individual ginseng saponins and balancing the composition of pharmacologically more active ginsenosides in Korean red ginseng saponin fractions enhanced the anti-inflammatory activity of the preparation* in vitro* (paper submitted, [32]). However, information on the effects of such preparations in arthritis is limited. In this study, we prepared Korean red ginseng saponin fraction-A (RGSF-A) from the dried roots ofKorean red ginseng. RGSF-A was further analyzed by HPLC for the identification of major seven PD- and three PT-ginsenoside components (Figure 1 and Table 1). The dominant chemical class in RGSF-A is PPD (87%) with the main 3 ginsenoside components Rb1, Rc, and Rb2 comprising of 37.4, 25.5, and 19% of the total PPD, respectively. We herein report that RGSF-A modifies inflammatory cell and cytokine imbalances and attenuates the severity of arthritis.

## 2. Materials and Methods

### 2.1. Preparation of Korean Red Ginseng Saponin Fraction-A (RGSF-A)

The preparation of RGSF-A was described previously [33]. Briefly, the dried roots ofKorean red ginseng were extracted with ethanol and the crude ginseng extract was air-dried at 60°C for 2 days followed by 3 times aqueous extractions of the crude extract powder at 95–100°C. The extract was then subjected to ultrafiltration in a membrane having a size of a molecular weight cutoff (MWC) 100,000 (Millipore, MA, USA). Finally, the filtrate was harvested concentrated via evaporation, freeze-dried, and designated as red ginseng saponin fraction-A (RGSF-A). RGSF-A was further analyzed by HPLC for the identification of major chemical components (Figure 1, Table 1).

### 2.2. Animals

Male DBA/1J mice (8-week age) were obtained from The Jackson Laboratory, (Maine, USA), and housed under specific pathogen free conditions with free access to feed and water. The experiments were conducted according to internationally accepted guidelines on the use of laboratory animals and the protocols were approved by the Institutional Animal Care and Use Committee (IACUC) of Daejeon University, Republic of Korea.

### 2.3. Collagen-Induced Arthritis

CIA was performed according to nature protocols [34]. Briefly, bovine collagen type II (bCII) (Chondrex, Inc., WA, USA) was dissolved in 10 mM acetic acid to 2 mg/mL. This solution was then emulsified in equal volumes of complete Freund's adjuvant (CFA) containing 250 *μ*g/mouse heat-killed mycobacterium tuberculosis H37Ra (Difco laboratories Inc., MI, USA). Then, 200 *μ*L of the emulsion was injected intradermal at the base of the tail of each mouse on day 0. On day 21, after primary immunization, mice were given booster doses of 200 *μ*L of CII in incomplete Freund's adjuvant (IFA).

### 2.4. Treatment

RGSF-A (with the composition in Table 1) was dissolved in dimethyl sulfoxide and freshly diluted in PBS for oral administration. Thirty mice were randomly allotted into five groups of 6 each. Group 1 was used as the nonimmunized (normal), whereas mice in groups 2–5 were immunized on day 0, followed by booster dose on day 21. Group 2 was immunized and untreated vehicle (phosphate-buffered saline (PBS)); group 3 was treated with methotrexate (MTX 0.2 mg/kg/day, intraperitoneal); groups 4 and 5 were treated with RGSF-A 150 and 50 mg/kg/day orally, respectively. RGSF-A treatment was initiated at 8 days after booster dose and given orally 5 days/week for 5 weeks.

### 2.5. Clinical Assessment of Arthritis

The clinical arthritis was assessed daily beginning from 15 days of primary immunization and arthritic score was conducted by three independent, blinded examiners for 3 times per week. The severity of arthritis in each of the four paws was graded in a scale of 0–4 according to the established protocol [34] and ranked as follows: 0: no evidence of erythema and swelling, 1: erythema and mild swelling confined to the tarsals or ankle joint, 2: erythema and mild swelling extending from the ankle to the tarsals, 3: erythema and moderate swelling extending from ankle to metatarsal joints, and 4: erythema and severe swelling encompass the ankle, foot, and digits, or ankylosis of the limb. Each limb was graded, giving a maximum possible score of 16 per animal.

### 2.6. Histological Analysis

For histologic analysis, hind paws were randomly collected from 4 mice of each group, fixed in 10% buffered formalin, and decalcified in 15% ethylenediaminetetraacetate (EDTA). The whole hind paw was paraffin embedded and 5 *μ*m sagittal serial tissue sections were processed before hematoxylin and eosin (H&E) stain according to the standard methods. Histopathological changes were microscopically examined and scored in a blinded manner by two independent observers based on cell infiltration, cartilage destruction, and bone erosion parameters. Histopathologic changes were also assessed and scored as graded (0 to 3) scoring system as follows: 0: normal joint structure, 1: mild changes, synovitis, and pannus front with few discrete cartilage focal erosions, 2: moderate changes, accompanying loss of large areas of cartilage, eroding pannus front, and synovial hyperplasia with infiltrating inflammatory cells, and 3: severe synovitis, cartilage and bone erosion, and destruction of joint architecture.

### 2.7. Flow Cytometry

Total cell counts from draining lymph nodes, joints and peripheral blood mononuclear cells (PBMCs) of vehicle, RGSF-A, or MTX treated mice were conducted before flow cytometric analysis was performed by counting absolute cell numbers manually in a hemocytometer chamber (Fisher, Pittsburg, PA, USA). Inguinal and axillary draining lymph node (dLN) cells of 4 arthritic mice from each treatment group were digested with collagenase type D (Roche) (1 mg/mL) and DNase I (Roche) (0.1 mg/mL) in Hanks' balanced salt solution at 37°C for 30 min and passed through mesh screens to prepare a single-cell suspension. The cells were washed, counted, and incubated with fluorochrome-conjugated antibodies for fluorescence-activated cell sorter (FACS) analysis. To obtain cells from arthritic joints, the limb joints of 5 mice from each treatment group were removed, and synovial tissue was isolated as described [35]. Briefly, the muscle, patella, and ligament were removed; the synovial tissue was isolated, cut into small pieces, and incubated with 1 *μ*g/mL type VI collagenase (Sigma) in Hanks' balanced salt solution (Sigma) with 2% FBS and 1 mM EDTA. The cells were harvested, washed, and incubated with antibodies for FACS analysis (FACSCalibur; BD Biosciences, San Jose, CA). The T- or B-lymphocyte subpopulations, neutrophils, and antigen-presenting cells were sorted using cell surface marker antibodies. Antibodies from eBioscience, Inc., (San Diego, CA, USA) used in this study included the following: anti-CD3-fluorescein isothiocyanate (FITC), anti-Alexa Fluor 488; anti-CD20-FITC, anti-B220- phycoerythrin (PE), anti-Allophycocyanin (APC); anti-CD8-PE, anti-CD4-PE, APC; anti-CD25-APC, anti-CD19-PE; anti-Gr-1-PE, anti-CD11b-FITC, anti-MHC-II-PE, anti-CD11c-APC, and anti-CD69-APC.

### 2.8. Analysis of Anticollagen Type II and Rheumatoid Factor- (RF-) IgM and IgG Antibody Productions

Vehicle-, RGSF-A-, or MTX-treated mice were bled at the termination of the experiment, and sera were analyzed for anti-CII IgG, RF-IgG, and RF-IgM antibody levels by quantitative ELISA. Briefly, ELISA plates (Thermo Fisher Scientific, NY, USA) were coated with 10 *μ*g/mL of type II collagen, RF-IgG, or RF-IgM dissolved in Tris buffer (50 M Tris, containing 200 mM NaCl, pH 7.4, 0.1% Tween 20), washed and blocked with 3% bovine serum albumin in Tris buffer, and then incubated with serial dilutions of test sera overnight at 4°C. After three washes, bound CII-IgG, RF-IgG, or RF-IgM were detected by incubation for 1 h with horseradish peroxidase-conjugated sheep anti-mouse IgG (BD Biosciences). After washing, plates were developed using ABTS (Roche Diagnostic Systems, IN, USA) as substrate, the reaction was stopped with 2 M H_2_SO_4_, and the absorbance was then measured at 450 nm in a Spectra Max Plus reader (R&D systems, Minneapolis, MN, USA). A standard serum from arthritic and nonimmunized syngeneic mice was added to each plate in serial dilutions as positive and negative controls, respectively.

### 2.9. Preparation of Spleen Cell Suspensions from CIA Mice

Spleens were aseptically removed from 4 mice in each group and washed with cold PBS and the tissues were minced. Single-cell suspensions were prepared by passing each spleen through a 70 *μ*m cell strainer by crushing with the end of a 10 mL plastic syringe plunger (BD Falcon; Bedford, MA, USA). The suspensions were then centrifuged on Ficoll-Paque TM at 1200 g for 5 min (Fisher Scientific, PA, USA) and the cell layers were collected. After repeated washes in PBS, the cells were resuspended in triplicate in 24-well plates cultured with RPMI 1640 medium containing 50 *μ*M 2-mercaptoethanol, 100 U/mL penicillin, 100 *μ*g/mL streptomycin, 50 *μ*g/mL gentamicin, and 10% fetal bovine serum (FBS). The cells were dispersed in trypsin, counted, and adjusted to 2 × 10^6^ cells/mL.

### 2.10. Analysis of Plasma and Cultured Primary Spleen Cell Cytokine Levels

For the analysis of serum and splenocyte cytokine levels, immunoassays were performed using antibodies against IL-1*β*, TNF-*α*, IL-6, IFN-*γ*, and IL-10 of a mouse cytokine immunoassay kit (R & D Systems, MN, USA). Blood samples were collected from all mice via cardiac punctures prior to sacrifice and allowed to clot for 2 h at room temperature and centrifuged at 2000 g for 20 min at 4°C to obtain serum. All measurements were made according to the instructions given by the manufacturers of the ELISA kits.

For the analysis of splenocyte cytokine levels, single-cell suspensions at a density of 2 × 10^6^ cells/well were cultured in triplicate to 24-well plates. After 24 h incubation, splenocytes in the absence or presence of RGSF-A (50 or 150 mg/kg) or MTX (0.2 mg/kg) were stimulated with 50 *μ*g/mL anticollagen type II peptide (cyanogen bromide cleaved peptide 11 (CB11) of bovine type II collagen (Chondrex, Inc., WA, USA)), for 48 h at 37°C in a 5% CO_2_-humidified atmosphere. Cytokine (IL-17, IL-6, and IL-10) levels in the culture media supernatants were assayed as indicated above in accordance with the manufacturer's recommendations.

### 2.11. Statistical Analysis

Significant changes in clinical arthritis as a result of drug treatment were determined using a dynamic modeling approach, assuming a linear fit for the slope of arthritis progression for each individual animal (SAS Institute, Inc., Cary, NC, USA, version 9.2). Significant differences in serum cytokines and antibody levels were assessed using the Student's *t*-test, and *P* < 0.05 was considered statistically significant. The clinical and histological score was analyzed using nonparametric analysis; Mann-Whitney *U* test was used when two groups were compared. For differences among study groups, we used the Kruskal-Wallis method followed by Dunn's test.

## 3. Result

### 3.1. Chromatographic Profile of Red Ginseng Saponin Fraction-A (RGSF-A)

High performance liquid chromatographic (HPLC) analysis of RGSF-A revealed that RGSF-A constitutes seven 20(*S*)-protopanaxadiol (PPD) (87. 4%) and three 20(*S*)-protopanaxatriol (PPT) (9. 6%) major chemical components. The PPD group is the dominant class in RGSF-A among which the 3 ginsenoside components Rb1, Rc, and Rb2 are comprising of 37.4, 25.5, and 19% of the total PPD, respectively (Table 1 and Figure 1) [33].

### 3.2. RGSF-A Treatment Reduces Severity of Collagen II-Induced Arthritis

We initiated RGSF-A treatment at the doses of 50 or 150 mg/kg after 8 days of booster dose and assessed the severity of arthritis according to arthritic score system described [34]. The arthritic index shown in Figure 1 indicated that arthritis was progressed rapidly in vehicle-treated mice. However, RGSF-A significantly decreased the severity of paw swelling and arthritis score in a dose-dependent manner (Figure 1(a)). MTX (0.2 mg/kg), which was used as a control to our test compound RGSF-A, strongly reduced the clinical score of arthritis.

### 3.3. RGSF-A Attenuates the Severity of Cellular Infiltration and Cartilage Erosion

**Figure 2:**
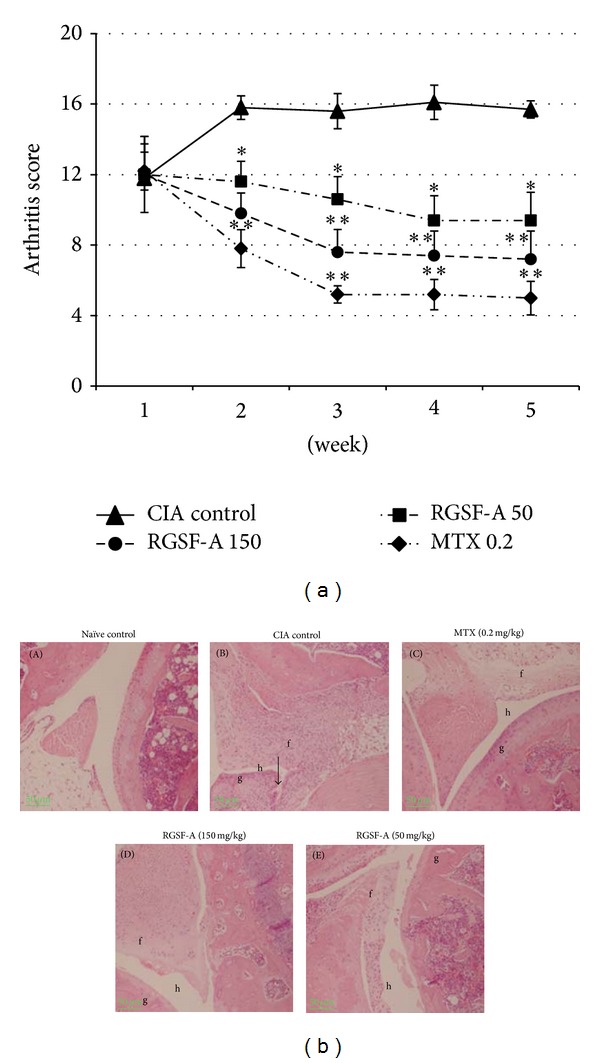
RGSF-A reduces the severity of clinical and histopathological signs of arthritis in CIA mice. (a) CIA was induced in DBA1J mice and 8 days after booster dose CIA mice injected either with phosphate-buffered saline (control) or 0.2 mg/kg methotrexate intraperitoneal or with oral dose of RGSF-A 50 or 150 mg/kg daily for 5 weeks. Arthritic score was assessed for the indicated weeks. (b) Histology of paw joints from CIA mice treated with vehicle, MTX, or RGSF-A. Paw joints were collected on day 54 after booster dose of naïve unimmunized (A), vehicle CIA mice (B), 0.2 mg/kg MTX (C), 150 mg/kg RGSF-A (D), or 50 mg/kg RGSF-A (E) treated CIA mice. Joints are formalin fixed, decalcified, sectioned, and stained with hematoxylin-eosin stains. Extensive leukocyte infiltration and synovial expansion into the articular surface are observed in vehicle-treated CIA mice (B), with massive inflammatory cell infiltration (f), synovial hyperplasia (g), narrowed articular space (j), and cartilage erosions (downward arrow). RGSF-A treated groups developed a less cellular infiltration (f) with a reduced hyperplasia (g) and clear articular space (h), which is comparable to MTX (D) groups. Histopathological images are magnified at 50x. Symbols in (a) represent mean value with SEM shown. *P < 0.05 and **P < 0.01, as compared with vehicle treatment; *n* = 6 for each group.

Since CIA and RA are well characterized with synovial hyperplasia, synovitis, pannus formation, and cartilage and bone erosion in the joint [1], we assessed the protective effects of RGSF-A on CIA induced arthritis by histological analysis of mice hind paws. Consistent with a reduced paw swelling observed in clinical score, a decreased histological sign of inflammation and cartilage erosion was observed. The vehicle-treated group (Figure 2(b), microphotograph B) had serious cellular infiltration (f), synovial hyperplasia (g), narrowing of articular space (h), and cartilage erosion (down arrow), whereas significantly diminished signs of the above indicated pathological lesions were observed in RGSF-A treated groups (Figure 2(b) microphotograph D and E). This suggests that RGSF-A administration reduces joint immune cell infiltration and inflammatory response of infiltrating/proliferating synovial cells.

### 3.4. RGSF-A Modulates Immune Cell Activation in Draining Lymph Node or Joint Cells

**Figure 3:**
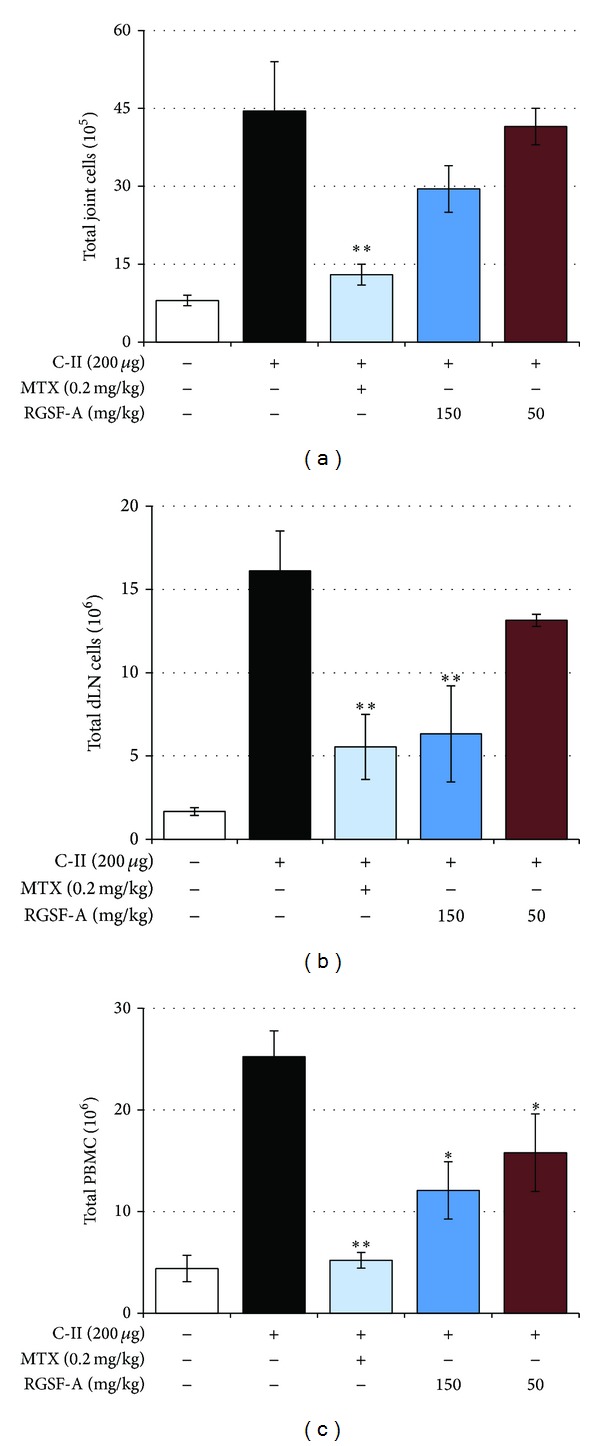
RGSF-A influences the total cell count of draining lymph nodes (dLN), joints, and peripheral circulation in CIA mice. Cells were counted manually using hemocytometer from synovial fluids (a), dLN (b), or peripheral blood mononuclear cells (PBMCs) (c) as indicated in Section 2. Bar graphs are summary of a triplicate count of 6 mice in each group. *P < 0.05 and **P < 0.01 as compared with vehicle treatment.

**Figure 4:**
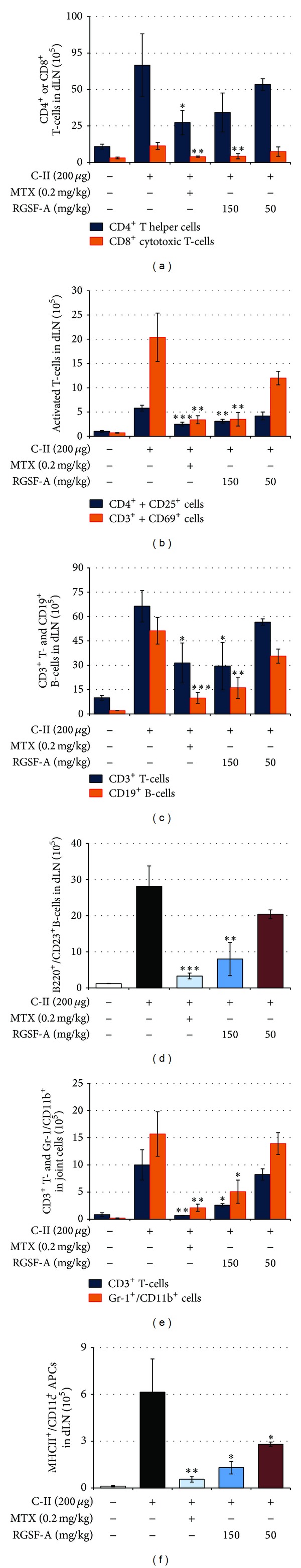
RGSF-A treatment affects CIA immune cell subpopulation activation and colocalization in dLN and joints of CIA mice. Flow cytometry was performed to detect cell surface molecules as indicated in the methods section. (a) CD4^+^ T helper (black bars) and CD8^+^ cytotoxic (gray bars) T-lymphocyte subpopulations are depicted. (b) Bar graphs show a relative subpopulation of CD4^+^CD25^+^ double positive T lymphocyte CD4 T-cells (black bars) and CD3^+^CD69^+^ double positive, early T lymphocyte activation marker (gray bars). (c) Relative colocalization of T-lymphocytes expressing CD3^+^ cell surface marker (black bars) and a subpopulation of B-lymphocytes with CD19^+^ cell surface markers (gray bars) were analyzed in mice treated with RGSF-A, MTX, or vehicle by flow cytometry. (d) CIA-stimulated B220^+^CD23^+^double positive cells as marker of active B-lymphocyte were analyzed. (e) T-lymphocytes expressing CD3^+^ surface markers (black bars) with neutrophils (gray bars) expressing Gr-1^+^CD11b^+^ surface markers infiltrated in the joints. (f) Antigen presenting cells expressing MHCII^+^CD11c^+^ double positive cell surface marker (black bars) were analyzed as cellular infiltrates in dLN. *P < 0.05, **P < 0.01, and ***P < 0.001 as compared with vehicle treatment; *n* = 6 for each group.

To better understand whether RGSF-A treatment influences the relative subpopulation and activation status of inflammatory cells from dLNs or joints during the onset of CIA, we performed multiparameter FACS analysis in mice after the disease progression. Total cell counts of joints, PBMCs and dLNs revealed that the cell population of CIA mice markedly increased compared to naive control (Figure 3). However, RGSF-A at higher dose (150 mg/kg) significantly reduced cell counts from the dLNs and PBMCs, respectively (Figures 3(b) and 3(c)). The same trend was also observed in joint cell count, although significant level was not achieved (Figure 3(a)). We also examined whether RGSF-A affects the distribution or differentiation of distinct T- and B-cells subsets. The relative population of CD4 (black bar) and CD8 (gray bar) T-lymphocytes proliferation was increased in dLN of CIA mice (Figure 4(a)). While the decrease in CD8 T-cells was significant in RGSF-A- or MTX-treated groups, the difference in CD4 T-cell subpopulations was not significant. Similarly, CD4/CD25 double positive (black bars), marker of activated or regulatory CD4 cells, and CD3/CD69 double positive (gray bar), early T-cell activation marker, subpopulations were markedly reduced by RGSF-A (150 mg/kg) or MTX treatment (Figure 4(b)). The relative population of T- and B-lymphocytes in the dLN of CIA mice was compared and RGSF-A at higher dose (150 mg/kg) significantly reduced T- (black bar) and B- (gray bar) cell populations (Figure 4(c)). In addition, CIA B-lymphocyte activation (increase in CD20/CD23 double positive cell subpopulation) was significantly decreased by high dose RGSF-A treatment (Figure 4(d)). As depicted in Figure 4(e), colocalization of CIA-activated CD3 expressing T-lymphocytes (black bars) and Gr-1/CD11b expressing granulocytes (neutrophils, gray bars) in joints was highly diminished in RGSF-A (150 mg/kg) or MTX group, suggesting the inhibitory effect of RGSF-A in leukocyte infiltration to the synovium, which indicates a beneficial therapeutic property of the test preparation. Similarly, CIA upregulation of MHCII/CD11c expressing antigen presenting cells (APCs) in the dLN was highly reduced in RGSF- or MTX-treated CIA mice (Figure 4(f)).

### 3.5. RGSF-A Inhibits Serum Levels of CIA Antibody Production

**Figure 5:**
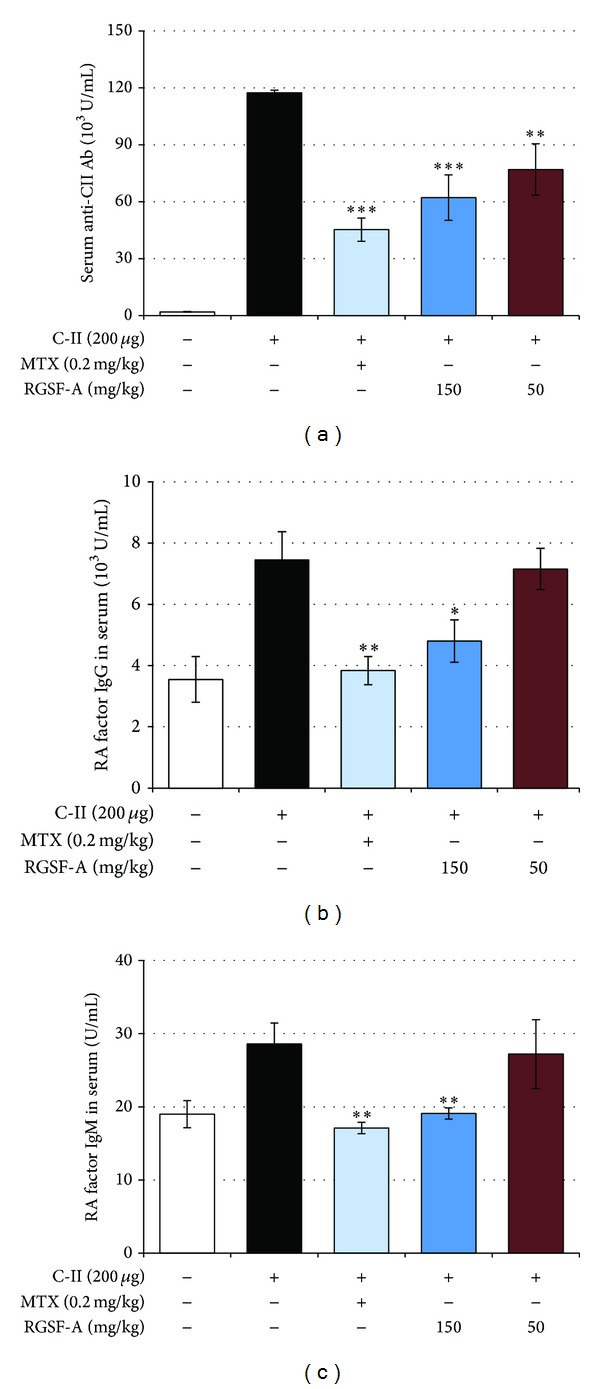
RGSF-A modulates CIA anti-CII IgG and rheumatoid factor (RF) serum antibody levels in mice. (a) Total anti-CII IgG antibody level was measured by enzyme-linked immunosorbent assay (ELISA) in mice treated with RGSF-A, MTX, or vehicle. The serum levels of RF IgG (b) and RF IgM (c) were determined by ELISA. *P < 0.05, **P < 0.01, and ***P < 0.001 as compared with vehicle treatment; *n* = 6 for each group.

In CIA mice, the activated dendritic cells (DCs) migrate to dLNs and present the processed CII peptide on appropriate MHC class II molecules to naïve T-cells. Engagement of the activated DCs and T-cells creates an environment in which adaptive immunity to CII is induced. As shown in Figure 5, RGSF-A treatment resulted in a significant suppression in anti-CII autoantibody production. The levels of total anti-CII IgG (Figure 5(a)), rheumatoid factor (RF) IgG (Figure 5(b)), and RF IgM (Figure 5(c)) were significantly decreased in RGSF-A- or MTX-treated groups. The result suggests that prolonged treatment of RGSF-A to mice with chronic arthritis resulted in a significant reduction in the serum levels of anti-CII IgG antibodies, which correlates with its effect in activated B-cells (Figure 4).

### 3.6. RGSF-A Modulates Serum and Cultured Splenocyte Cytokine Levels from CIA Mice

**Figure 6:**
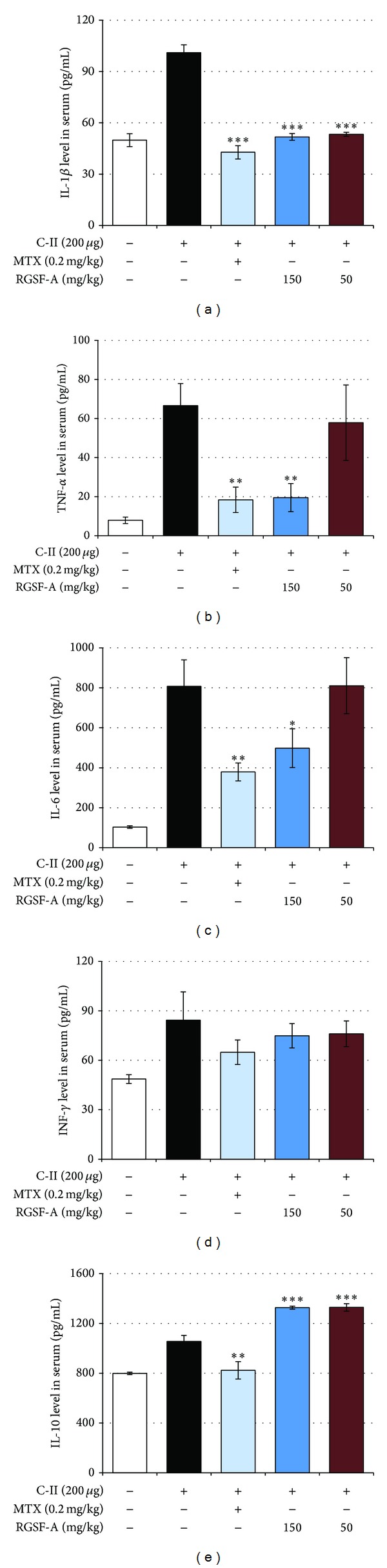
RGFS-A modifies serum levels of pro- and anti-inflammatory cytokines in CIA mice. CIA mice were treated with the indicated doses of RGSF-A, MTX, or vehicle. Blood was drawn by cardiac puncture from CIA mice at the end of the experiment and serum cytokine levels were measured by ELISA using protocols supplied by the manufacturer. Arthritis-induced IL-1*β* (a), TNF-*α* (b), IL-6 (c), INF-*γ* (d), and IL-10 (e) levels in the serum of CIA mice were analyzed. *P < 0.05, **P < 0.01, and ***P < 0.001 versus vehicle control; *n* = 6 for each group.

Levels of proinflammatory and anti-inflammatory cytokines in serum as well as splenocytes isolated from CIA mice and stimulated with anti-C-II peptide were analyzed using a multiplex immunoassay at the termination of the experiment. Consistent with the joint swelling, IL-1*β*, TNF-*α*, and IL-6 in the vehicle-treated CIA mice were systemically increased in serum. The elevated cytokine levels in mice treated with RGSF-A at 150 mg/kg, however, were decreased progressively in a manner correlated positively with the degree of joint swelling in individual animals (Figures 6(a), 6(b), and 6(c)), respectively. On the other hand, RGSF-A did not affect serum levels of IFN-*γ*, while it significantly increased IL-10 levels (Figures 6(d) and 6(e)), suggesting the shift of Th1/Th2 balance towards the anti-inflammatory cytokine signature Th2 cells that would be expected to be clinically beneficial.

**Figure 7:**
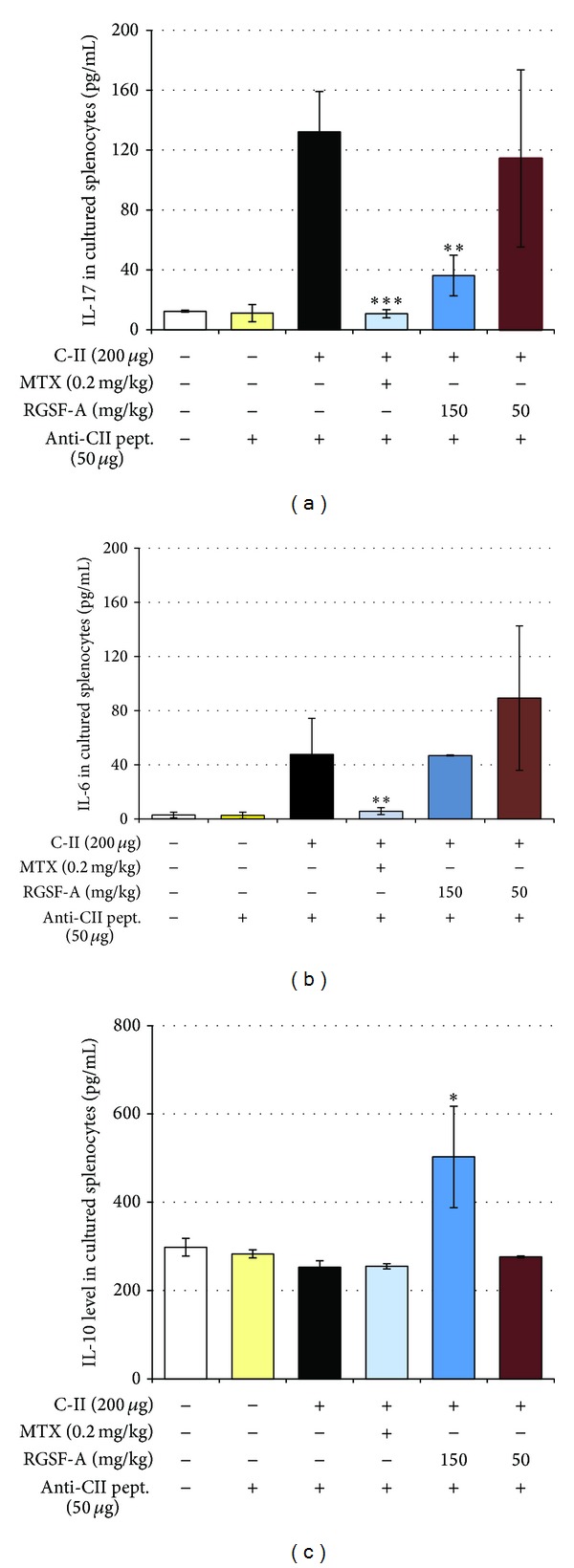
Effect of RGSF-A on cytokine production profile in cultured and anti-CII peptide stimulated splenocytes isolated from CIA mice. Splenocytes from naïve, RGSF-A, MTX, or vehicle-treated mice isolated, cultured, and collagen peptide stimulated followed by analysis of cytokine levels in the supernatant as indicated in the materials and methods section. Treatment of RGSF-A at higher dose reduced CIA IL-17 (a) and IL-6 (b) secretions but induced IL-10 production in splenocytes from CIA mice cultured with anti-CII peptide. *P < 0.05, **P < 0.01, and ***P < 0.001 as compared with vehicle treatment; *n* = 6 for each group.

Since an imbalance between pro- and anti-inflammatory cytokine activities favors the induction of autoimmunity, chronic inflammation, and thereby joint damage [3], we in addition to serum levels also examined (Figure 6) whether such an imbalance could be modulated by RGSF-A treatment in cultured and C-II peptide stimulated splenocytes isolated from CIA mice. As depicted in Figure 7, RGSF-A significantly decreased the secretion of proinflammatory cytokines including IL-17 and IL-6 (Figures 7(a) and 7(b)). Interestingly, RGSF-A promoted the anti-inflammatory cytokine IL-10 production (Figure 7(c)). This suggests that RGSF-A may modulate the imbalance of cytokines within a complex regulatory network related to specific immunological processes that promote autoimmunity, chronic inflammation, and tissue destruction.

## 4. Discussion

In this study, we prepared RGSF-A, a saponin fraction of ginsenosides from Korean red ginseng, with the higher proportion of 20-(S) protopanaxadiol group (87.3%) containing the three dominant components (Rb1 (37%), Rc (26%), and Rb2 (19%)) and determined its antiarthritic activity in mouse model of bovine type-II collagen-induced rheumatoid arthritis. We herein provide evidence that RGSF-A shows a highly effective alternative treatment for collagen-induced arthritis in mice. Daily oral dosage of RGSF-A decreased the paw swelling and severity of arthritis and ameliorated joint damage. The therapeutic effect of this saponin preparation is associated with a striking reduction of two deleterious components of the disease, the inflammatory and autoimmune responses.

At the oral doses of 50 and 150 mg/kg, RGSF-A revealed a strong antiarthritic property capable of reducing CIA paw edema and swelling as observed in arthritis score. Histologically, we noted significant attenuation in cellular infiltration of the synovium, synovial hyperplasia, and cartilage damage in RGSF-A-treated mice. The present study appears to be the first report demonstrating the possible antirheumatic effects of ginseng saponin fraction mainly consisting of three ginsenosides (Rb1, Rc, and Rb2), which could reduce the disease progression and joint destruction with a pattern comparable to that of MTX, the first-line drug used in the management of RA. From a therapeutic point of view, it is important to take into account the ability of delayed administration of RGSF-A to ameliorate ongoing disease, which is an essential prerequisite for an antiarthritic agent, as treatment was started 8 days after the booster doses. Besides, the fact that we did not observe long-term treatment side effects with time suggests that RGSF-A as a food drug may be alternative therapy that could induce remission of the disease in prolonged treatment regimen.

Even though progress has been made in the advancement of RA therapy ranging from conventional to biological, many questions remain with respect to partial response to the treatment of available therapies, and disease remission is achieved in only a minority of patients [8–10]. Therefore, a demand for alternate inexpensive agents with improved therapeutic potential and minimal side effects exists, and there is still a need to develop novel alternative antirheumatic therapies.

On the other hand, an interest is recently arising on natural products, which possess minimal side effects and are inexpensive to be considered as alternative treatment options in arthritis. Khanna et al. reviewed such anti-inflammatory agents derived from plants that include flavonoids, terpenes, quinones, catechins, alkaloids, anthocyanins, and anthoxanthins [11]. More recently, pomegranate extract [12], epigallocatechin-3-gallate [13], resveratrol [14], curcumin [15], and other natural active ingredients [11] have been reported to suppress multiple inflammatory and catabolic molecular mediators of arthritis and this report in our study adds to the available information on the potential of natural products to be alternative antiarthritic agents.

The demonstrations in the present study revealed that RGSF-A strongly reduces the inflammatory response during CIA progression by downregulating the T-lymphocyte activations (early (CD3/CD69), cytotoxic (CD8 T-cells), and mature CD4 (CD4/CD25)) in dLNs. Besides, RGSF-A downregulates both immature (CD19) and mature-activated (CD20/CD23) autoreactive B-cells and activated (MHCII/CD11c) DCs in dLN of CIA mice. A similar trend of reduced inflammatory response was reflected on the compound's ability to downregulate T-lymphocyte and neutrophil (Gr-1/CD11b) activations in the joints of CIA mice. Thus, RGSF-A did influence the subpopulation and activation of T- and B-lymphocytes, DCs, and neutrophils in dLN or joint during CIA. These results suggest that our ginseng saponin preparation with known ginsenoside compositions may play an essential role in limiting lymphocyte and myeloid cell trafficking. The observed suppressive effect of RGSF-A in the above indicated inflammatory cell subpopulations was strongly correlated with its inhibitory effect on CIA serum levels of total anti-CII IgG, RF-IgG, and IgM antibodies. It is likely that the suppressive effect of RGSF-A on anticollagen antibody production observed in our studies may be due to inhibitory effects on immune cell trafficking. CD4 T-cells are reported to express CD69, an early marker of activation that correlates with disease severity, and produce CD40 ligand, a member of the TNF receptor (TNFR) superfamily responsible for the promotion of B-cell proliferation, immunoglobulin production, monocyte activation, and dendritic cell differentiation [4]. In addition, the CII-specific antibodies appear to have the potential to initiate an articular inflammatory response [36, 37], and activation of the complement cascade especially C5a (a cleavage product of C5) by anti-CII antibodies recruits neutrophils and macrophages. The recruited neutrophils and macrophages are then activated to secrete chemotoxic substances and proinflammatory cytokines (IL-1*β*, TNF-*α*, IL-8, and IL-6), NO, and PGE2 [36]. Therefore, attenuation of the above indicated pathologic features of CIA by RGSF-A makes it a potential candidate to be considered in RA therapeutic strategy.

In our study, RGSF-A downregulated the proinflammatory cytokine productions including TNF-*α*, IFN-*γ*, IL-6, IL1*β*, and IL-17 in dLN and cultured splenocytes, while it elevated the levels of the anti-inflammatory cytokines IL10. The capacity of RGSF-A to regulate a wide spectrum of inflammatory mediators might offer a therapeutic advantage over other treatments directed against a single mediator, such as the new biological agents. And the fact that RGSF-A treatment enhanced IL10 production in isolated splenocytes suggests a shift towards Th2 responses in RGSF-A-treated groups. In line with this study, cytokines are implicated in each phase of the pathogenesis of rheumatoid arthritis, by promoting autoimmunity (during the prearticular phase), by maintaining chronic inflammatory synovitis and by driving the destruction of adjacent joint tissue [3]. IL10 and IL-4 have been recognized as signature cytokines of Th2 cells that exert regulatory functions and are involved in the restoration of the immune tolerance [38]. Cytokines, therefore, integrate the immunoregulatory and tissue-destructive events that underlie the clinical presentation and progression of rheumatoid arthritis, and thus those therapeutic agents regulating the cytokine profiles in RA are believed to be the drugs of choice [39]. The fact that RGSF-A inhibited the production of proinflammatory but enhanced anti-inflammatory mediators in splenocytes isolated from CIA mice on* in vitro* CII-specific response also supports the direct effect of RGSF-A in cytokines regulation. This suggests that, in addition to the reduction in inflammatory infiltration, RGSF-A also attenuates the inflammatory response. This study does not rule out the possibility that global immunomodulation of RGSF-A may also be associated with its influence on the activities of mucosal microbiota, which needs to be addressed.

Our findings in the present study suggest that further identification of the specific molecular target (s) of the 3 individual ginsenosides (Rb1, Rc, and Rb2), which could mediate* in vivo* anti-inflammatory property, needs to be further explored. Together these data raise the possibility of a new generation of orally available therapeutic agent. The ideal therapeutic strategies should include a series of parameters including genetic, metabolomic, and transcriptomic profiles and soluble inflammatory makers together with markers of articular damage most of which are evaluated and attenuated by RGSF-A in this study.

One of the RGSF-A constituents, ginsenoside Rc, suppressed the molecular complexes formation between p38 and TBK1 enzymes and reduced the activation of their downstream substrate proteins and limited the release of inflammatory cytokines, suggesting anti-inflammatory and antirheumatic role of the compound(s) (paper submitted, [32]). In agreement with these data, inhibiting upstream kinases such as TBK1 and the activation and translocation of AP-1 and IRF-3 are potential therapeutic targets for rheumatoid arthritis [40–42].

From the structural-activity relationship point of view, the presence of arabinose linked at the glucopyranosyl group may have enhanced the anti-inflammatory activity of ginsenoside Rc. This is in line with previous reports suggesting that antioxidant and anti-inflammatory activities of ginsenosides depend upon their sugar moieties and linkage positions, the types of aglycone, and total number of hydroxyl groups [43–45]. In particular, ginsenoside Rc has previously been reviewed to inhibit intracellular reactive oxygen species and the radical scavenging activity better than other ginsenosides [43].

## 5. Conclusions

RGSF-A treatment reduced arthritis score and histological lesions, attenuated immune and inflammatory responses, modified cytokine imbalances, and improved the severity of arthritis. This effect was linked to the three main ginsenoside components of RGSF-A, and ginsenoside Rc was particularly involved in inhibition of TBK1 activation and modulates downstream signaling pathway. This work suggests RGSF-A as a new immunomodulatory and anti-inflammatory agent with the capacity to ameliorate the inflammatory response at multiple levels. This provides a powerful rationale for the additional assessment of the efficacy of the ginsenosides Rb1, Rc, and Rb2 as a novel therapeutic agent to the treatment of RA and other chronic autoimmune disorders as either a combined therapy or an individual entity.

## Figures and Tables

**Table 1:** Chemical composition of RGSF-A.

Group	RGSF-A components	Extract composition (mg/g) or (%)
20(*S*)-Protopanaxadiol		**422.6 (42.3)**
	Rb1	158.0 (15.8)
	Rb2	80.0 (8.0)
	Rb3	12.2 (1.2)
	Rc	107.6 (10.8)
	Rd	39.6 (4.0)
	F2	16.4 (1.6)
	Rg3	11.8 (1.2)
20(*S*)-Protopanaxatriol		**95 (9.5)**
	Re	33.6 (3.4)
	Rf	25.6 (2.6)
	Rg1	35.8 (3.6)

Total		520.6 (52)
